# Methyl Jasmonate: Putative Mechanisms of Action on Cancer Cells Cycle, Metabolism, and Apoptosis

**DOI:** 10.1155/2014/572097

**Published:** 2014-02-06

**Authors:** Italo Mario Cesari, Erika Carvalho, Mariana Figueiredo Rodrigues, Bruna dos Santos Mendonça, Nivea Dias Amôedo, Franklin David Rumjanek

**Affiliations:** Laboratório de Bioquímica e Biologia Molecular do Câncer, Instituto de Bioquímica Médica, Universidade Federal do Rio de Janeiro, Avenida Carlos Chagas Filho 373, Prédio CCS, Bloco E, Sala 22, Ilha do Fundão, Cidade Universitária, 21941-902 Rio de Janeiro, RJ, Brazil

## Abstract

Methyl jasmonate (MJ), an oxylipid that induces defense-related mechanisms in plants, has been shown to be active against cancer cells both *in vitro* and *in vivo*, without affecting normal cells. Here we review most of the described MJ activities in an attempt to get an integrated view and better understanding of its multifaceted modes of action. MJ (1) arrests cell cycle, inhibiting cell growth and proliferation, (2) causes cell death through the intrinsic/extrinsic proapoptotic, p53-independent apoptotic, and nonapoptotic (necrosis) pathways, (3) detaches hexokinase from the voltage-dependent anion channel, dissociating glycolytic and mitochondrial functions, decreasing the mitochondrial membrane potential, favoring cytochrome *c* release and ATP depletion, activating pro-apoptotic, and inactivating antiapoptotic proteins, (4) induces reactive oxygen species mediated responses, (5) stimulates MAPK-stress signaling and redifferentiation in leukemia cells, (6) inhibits overexpressed proinflammatory enzymes in cancer cells such as aldo-keto reductase 1 and 5-lipoxygenase, and (7) inhibits cell migration and shows antiangiogenic and antimetastatic activities. Finally, MJ may act as a chemosensitizer to some chemotherapics helping to overcome drug resistant. The complete lack of toxicity to normal cells and the rapidity by which MJ causes damage to cancer cells turn MJ into a promising anticancer agent that can be used alone or in combination with other agents.

## 1. Introduction

Cancer cells do not resemble normal cells in terms of morphology and metabolic behavior [1]; under this premise, much effort is invested worldwide in order to develop anticancer therapies that can kill cancer cells without harming normal cells. These therapies attempt to target differentially expressed functional molecules in cancer and normal, nontransformed cells. For this purpose, a myriad of new small molecular weight synthetic and/or natural inhibitor compounds are being tested aiming at achieving selective anticancer clinical treatments. Small molecular weight chemicals from plants (phytochemicals) often accomplish multitargeted anticancer activities including cell cycle arrest, inhibition of cell growth, proliferation, and metastasis and promote apoptosis and cell death [2].

**Figure 1:**
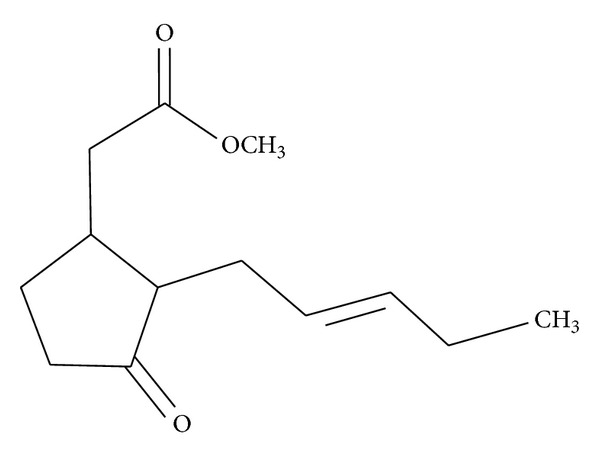
Chemical structure of methyl jasmonate (cyclopentaneaceticacid, 3-oxo-2-(2-penten-1-yl)-, methyl ester).

Methyl jasmonate (MJ), our focus in this review, is a natural cyclopentanone lipid (Figure 1) belonging to the jasmonates (JAs) family of plant oxylipin stress hormones (oxygenated fatty acids). JAs originate from *α*-linolenic acid released from the lipid pool of plant cells by lipases and are subsequently oxygenated by lipoxygenases (LOXs) to form hydroperoxide derivatives (cyclopentanones) [3–5]. The JAs family members consist mainly of jasmonic acid (JA), *cis*-jasmonate (CJ), and MJ [6]. They occur ubiquitously in plants and their production is induced by different types of environmental abiotic (UV radiation, osmotic stress, and temperature variation) and biotic (pathogens, predators) stresses, mediating signaling that triggers defense responses against those stresses [7]. When JAs are synthesized in response to stress, they induced the production of reactive oxygen species (ROS) and of plant secondary metabolites such as, for instance, phenolic compounds which are used as chemical defenses [6–12]. For instance, ultraviolet- (UV-) B radiation triggers JA accumulation [10, 13], whereas MJ is produced under herbivore attack and can act as a volatile signal recognized by neighboring plants to induce the synthesis of protease inhibitors [8]. JAs alter plant mitochondrial function and induce reactive oxygen species (ROS) leading to plant cell death in a way similar to mammalian apoptosis [12, 14].

MJ has been shown to have powerful anticancer activity through multiple mechanisms [15–24]. The purpose of this review is to gather most relevant findings concerning MJ-mediated anticancer activities in an attempt to integrate them into a functional view that could help us to develop new anticancer research strategies and therapies.

## 2. Effects of Jasmonates on Mammalian Cancer Cells

In 2002, Fingrut and Flescher [15] discovered that MJ strongly and adversely affected human lymphoblastic leukemia cells by suppressing their proliferation and inducing cell death, while nontransformed lymphocytes and normal keratinocytes were unaffected [15]. In these initial studies, they showed that JAs selectively targeted cancer cells in an *ex vivo* mixed population of leukemic and normal peripheral blood mononuclear cells (PBMCs) from a patient with chronic lymphocytic leukemia (CLL). JAs also increased the life span of T-cell lymphoma-bearing mice [17]. Thereafter JAs, including MJ and related synthetic analogs, were found to inhibit *in vitro* cancer cell proliferation and to induce cell death in other human and murine cancer cell types [16, 19–24], including human breast [15, 25], cervix [26–29], colon [30, 31], colorectal [32], gastric [33], hepatoma [34, 35], lung [19, 36, 37], lymphoma [15, 17, 18, 38], melanoma [15, 30, 39, 40], myeloid leukemia [41, 42], neuroblastoma [43–45], prostate [15, 46–48], and sarcoma [49] cancer cells (Table 1). Other results have shown that JAs and their synthetic derivates exerted selected cytotoxic effects *in vivo* towards metastatic melanoma [21, 39] and inhibited angiogenesis at high doses (it was the reverse at low doses) in the chorioallantoic membrane (CAM) of chicken embryo [40] (Table 2). In general, MJ has been found to be superior to CJ and JA in terms of cytotoxicity and induction of apoptosis in human cancer cells [33, 38, 44]. Independently if jasmonates are dissolved in an organic solvent or not, most experiments *in vitro* with JAs and MJ have been reported to exert their biological effects at similar low millimolar (mM) concentrations (Tables 1 and 3) excepting few cases where MJ and particularly some of its chemical derivatives were active at micromolar (*μ*M) concentrations *in vivo* (Table 2). Differential dispersion and/or availability of small hydrophobic MJ lipid droplets after phase separation in culture media or solubility in biological fluids *in vivo* might explain these differences. Nonetheless, JAs have been found to be nontoxic at doses higher than the usual pharmacological doses employed for other compounds (nM, µM); for instance, an i.v. injection of 236 mg MJ/kg body weight in mice (equivalent to *≈*5.0 mM circulating conc. in a 25 g adult mouse) was not toxic [15]. The nontoxic i.v. dose was, however, found lower by Reischer et al. in mice (75 mg/kg) [39]. On the other hand, oral acute toxicity studies performed with MJ on 10 Sherman Wistar rats reported a LD_50_ > 5 g/kg, showing a normal necropsy analysis, whereas the dermal acute dose was LD_50_ > 2 g/kg from studies in 10 albino rabbits, showing again a normal necropsy analysis [50]. No skin irritation was observed when MJ was topically applied at 2 g/kg to 10 albino rats and at 1% MJ in 6 albino New Zealand rabbits; similarly, there was no skin irritation when a 10% MJ solution was topically applied on 10 guinea pigs and on 50 volunteers (the latter assessed by the Human Repeat Insult Patch Test) [50]. Repeated application of the 10% MJ solution at the same skin site in guinea pigs and in 50 volunteers did not show dermal sensitization; neither photoirritation nor photoallergy was observed in the volunteers. No mucosal irritation was observed after a volume of 0.1 mL of neat MJ was instilled into the right eye of 6 white New Zealand rabbits (left eye served as control) [50]. Finally, when topically applied on cancerous and precancerous skin lesions of eight patients with different pathologies, MJ showed promising results: 3/8 patients with oral lichen planus exhibited positive responses, and 1/8 patient with leukoplakia had complete recovery (for 18 months following the first treatment), while MJ treatment of one patient with lentigo maligna of the face resulted in dry tumor surface with reduction of the metaplastic area during treatment, but the cancer reappeared three months later. MJ did not cause significant local or systemic side effects [51] (Table 2). The notable absence of toxicity of MJ to normal cells and to healthy human and animals at mM doses indicates that MJ may be used safely in cancer treatments at these doses [15, 19, 21, 22, 38, 39, 50]. Furthermore, based in toxicity studies, an exemption from the requirement of a tolerance for MJ was recently published by the US Federal Environmental Protection Agency (EPA) [52]. In this document, MJ was considered as a ubiquitous and naturally occurring plant hormone, regarded as a safe and natural part of the human diet through commonly consumed fruits. No toxicological endpoints were found for MJ through any route of exposure and stated that MJ is virtually nontoxic to humans and other nontarget organisms, through all routes of exposure, including oral. As further support for these arguments, it was mentioned that MJ was also assessed and approved by Food and Agriculture Organization/World Health Organization (FAO/WHO) as a food additive. Toxicity results reported in the EPA document [52] were found to agree with those previously published by other groups [50, 51].

### 2.1. Jasmonates (MJ) Can Cause Cancer Cell Cycle Arrest at Different Phases in Different Cancer Cell Types, Inhibiting Growth and Proliferation

The eucaryotic cell cycle is divided into four phases: G_1_, S, G_2_, and M that occur in response to growth factors or mitogens. The DNA synthetic (S) and mitotic (M) phases are preceded by gap phases (G_1_, G_2_). Chromosome duplication occurs during S phase; replicated chromosomes are segregated into individual nuclei (mitosis) during M phase and the cell then splits in two. Cell cycle progression can be regulated at G_1_, G_2_ points by various intracellular and extracellular signals. If extracellular conditions are unfavorable, cells delay progress through G_1_ and may enter a quiescent state known as G_0_, in which they can remain for days, weeks, or years before resuming proliferation. If extracellular conditions are favorable and signals to grow and divide are present, cells in early G_1_ or G_0_ progress through a restriction checkpoint late in G_1_ after which cells are committed to DNA replication even if the extracellular signals that stimulate cell growth and division are removed [53, 54]. Cell cycle activity is modulated by intracellular ROS levels and regulates cell survival, cell proliferation, and apoptosis [53–58]. The progression through the G_1_, S, G_2_, and M phases is promoted by cyclin-dependent kinases (CDKs), which are positively and negatively regulated by cyclins and cyclin kinase inhibitors (CKis), respectively, and by ROS. Unlike the passage through the S, G_2_, and M phases, G_1_ progression normally relies on stimulation by mitogens and can be blocked by antiproliferative factors; passage through the restriction checkpoint and entry into S phase is controlled by CDKs that are sequentially regulated by cyclins D, E, and A [53, 54, 56–58]. In general, CDK activity requires cyclin binding and depends on both positive and negative regulatory phosphorylations [54]. ROS levels influence the phosphorylating and ubiquitinating enzyme activites, thus controlling cell cycle progression [54, 57, 58].

A master gene regulating cell passage through the cycle is the Myc family of transcription factors; it switches on the simultaneous synthesis of thousands of different proteins required for cell growth and cell cycle function [58, 59]. To counteract Myc protein activity, the cell cycle is also regulated by the p53 protein [60, 61], a DNA-binding transcription factor that senses DNA damage (occurring after exposure to ionizing radiation, UV light, oxidative stress, or other DNA damaging agents) and activates genes to halt cell cycle progression in G_1_ for repair, or otherwise initiates apoptosis [56, 62]. When the damage caused by cell stress is mild, p53 activates mechanisms for cell cycle arrest and repair damaged DNA; if the damage is irreparable, p53 initiates apoptosis [63]. In case of mild cellular stress, low levels of p53 can activate the TP53-induced glycolysis and apoptosis regulator (TIGAR) gene that decreases the rate of glycolysis and hence ROS levels in the cell [64]. Glycolysis is essential for cancer progression, but TIGAR provides protection from ROS and apoptosis. However, if damage caused by stress is irreparable, p53 prevents proliferation of cells with altered DNA and induces increased levels of ROS to trigger cell death [56]. Increased levels of active p53 inhibit the cell entry into the S-phase or induce apoptosis [56]. Loss or inactivation of p53 results in loss of cell-cycle arrest or inhibition of apoptosis after DNA damage or physiological stress [65] leading to increased genetic instability, increased accumulation of mutations, and ultimately oncogenesis [61]. When the cell cycle is no longer regulated by p53, cells remain cycling (supported by *c*-Myc overexpression), subverting cell cycle exit, maturation, and terminal differentiation [66]. Over 50% of all human cancers harbor mutations and genetic alterations in cyclins and p53 genes directly affecting the function of critical cell cycle proteins, rendering the *p53* gene ineffective in mediating cell growth arrest and promoting cancer cell death [62, 67, 68]. ROS production is under p53 regulation, and in turn p53, being a redox-sensitive protein, is influenced by ROS levels; thus, ROS can act as both an upstream signal that triggers p53 activation and as a downstream factor that mediates apoptosis [69]. Thus, mutations in the *p53* gene downregulate its activity and, consequently, ROS production [70]. Through ROS, p53 can also directly control metabolic traits of cells [71], regulate mitochondrial membrane potential (Δ*ψ*_*m*_), and induce cytochrome *c*-independent apoptosis (blocked by Bcl-2); ROS-mediated disruption of Δ*ψ*_*m*_ constitutes a pivotal step in the apoptotic pathway of p53, and this pathway does not involve cytochrome *c* release [72]. ROS can thus regulate cell fate through p53, in a way that physiological ROS levels trigger cell protective pathways, while under cytotoxic oxidative stress p53 behaves more like a cell killer [70, 73].

JAs (mainly MJ) have been found to arrest cell cycle of different cancer cell types at different phases (Table 1): G_0_/G_1_, in human acute lymphoblastic leukemia Molt-4 [14, 38] and human neuroblastoma SK-N-SH, BE(2)-C [45]; G_0_/G_1_-S in human breast MCF-7 and melanocytic MDA-MB-435 cancer cells [25, 74]; S, in human neuroblastoma BE(2)-C [43]; S-G_2_/M, in human adenocarcinoma colon HT-39 [31]; G_2_/M, in nonsmall cell lung cancer (NSCLC) lines A549 and H520 [37], human neuroblastoma SH-SY5Y [44], human cervical carcinoma, and HeLa cells [27]. At 0.1 mM, MJ arrested *in vitro *human umbilical vein endothelial cells (HUVEC) at G_1_ [40].

The molecular mechanisms underlying the above jasmonate-mediated cell cycle arrest in cancer and embryonic HUVEC cells *in vitro* are not yet completely understood. Checking the above data against the UMD TP53 mutation database (http://www.p53.fr/) and revising the article by Berglind et al. [75], we could not find correlation between a given MJ-mediated phase arrest of cell cycle and the TP53 wild type or mutated status of a cancer cell type (data not shown), a result that would agree with the original observation of Fingrut and Flescher [15] stating that MJ-mediated cell death is independent of the p53 status [17]. However, other studies suggest that MJ could be acting by interfering different cell cycle regulatory mechanisms. In plants, MJ is able to bind the TGACCG motif which is present in cell cycle cyclin promoters [76]; we might speculate MJ recognition of TGACCG-like motifs in mammalian cells cyclin promoters; however, this would need experimental confirmation. In some plants, MJ impairs the G_2_-to-M transition by repressing M-phase gene activation [77]. MJ impaired the G_2_-to-M transition in the human neuroblastoma SH-SY5Y cells, downregulating the expressions of the proliferating cell nuclear antigen (PCNA, a processivity factor encircling DNA at sites of replication and repair) and N-Myc [45]. As mentioned above, *MYC* is the master gene allowing cancer cells to move along the cell cycle and regulating most genes needed to meet the metabolic demands for cell proliferation and survival [78]. Cancer cell cycle arrest at different phases is in itself closely dependent on the cell energy and as such it is extremely dependent on metabolic ATP availability and its use by hexokinase. Downregulation of Myc by MJ [45] is interesting result that must be confirmed, particularly because there is a lack of specific inhibitors against Myc [57].

In some cases of cell cycle arrest, MJs have been found to upregulate p21 [37] whereas in others to downregulate survivin mRNA and protein levels [43–45] (Table 1). Survivin is an important member of the inhibitor of apoptosis protein (IAP) family that also regulates entrance of the cell cycle to the M phase and is overexpressed in cancer cells acting linking both regulation of the cell cycle and inhibition of apoptosis [79].

### 2.2. Jasmonates (MJ) Can Cause Cancer Cell Death by Inducing Apoptosis and/or Necrosis

Apoptosis can be initiated by two signaling cascades, namely, the extrinsic and the intrinsic pathway. The classical extrinsic pathway is triggered by the binding of extracellular signals like tumor necrosis factor (TNF), tumor necrosis factor-related apoptosis-inducing ligand (TRAIL), and apoptosis antigen-1 (Fas) ligand (FASL) to death receptors such as tumor necrosis factor receptor 1 (TNFR1) and FAS [80]. This interaction leads to procaspase-8 activation which cleaves itself into caspase-8. Then, caspase-8 can cleave and activate procaspase-3 into caspase-3 that cleaves many substrates inside the cell inducing the typical morphological changes seen in apoptosis. The intrinsic pathway can be triggered by intracellular stress signals, such as oxidative stress and DNA damage. The diverse signals are sensed by mitochondria through the balance between pro- and antiapoptotic members of the Bcl-2 family of proteins. When proapoptotic signals overcome antiapoptotic signals, mitochondria become permeabilized leading to dissipation of the mitochondrial electrochemical potential, release of intermembrane proteins like cytochrome *c*, Smac/DIABLO, AIF, and HTRA2/OMI. Cytochrome *c* interacts with procaspase-9 and Apaf-1 to form the apoptosome complex that leads to caspase-3 activation [81]. The extrinsic pathway can connect with the intrinsic pathway through the cleavage by caspase-8 of Bid into tBid (truncated Bid), which is a proapoptotic member of the Bcl-2 family and can lead to mitochondria permeabilization [82, 83]. The members of the Bcl-2 family regulate mitochondria permeabilization and comprises antiapoptotic (Bcl-2, Bcl-xL), proapoptotic (Bax, Bak), and proapoptotic BH-3 only proteins (Bad, Bid, Puma, and Noxa) [82–84]. One of the mechanisms of mitochondria permeabilization is controlled by Bax and Bak proteins that are normally expressed in every cell but are maintained inhibited by the antiapoptotic members. When there is a signal to apoptosis, BH3-only proteins interact with the antiapoptotic members, releasing Bax/Bak or directly activating them, leading to their oligomerization and to mitochondrial outer membrane permeabilization (MOMP). Another mechanism for mitochondria permeabilization is the formation of the mitochondrial permeability transition (MPT) pore which is composed by the voltage-dependent anion channel VDAC, the adenine nucleotide translocase (ANT), and cyclophilin D [85]. The MPT pore, comprising VDAC1, is directly regulated by Bad [86]. In contrast, VDAC2 has been identified as an antiapoptotic, negative regulator of Bak [87–89].

One of the hallmarks of cancer is resistance to cell death [90]. This resistance is acquired by a host of different genetic defects in cancer cells, such as those present in the *p53* tumor suppressor gene [62, 68, 91]. Normal p53 responds to genotoxic, oncogenic, and other stress signals by inducing antiproliferative transcriptional programs leading to growth arrest (for cell repair) or apoptosis [92]. p53 mediates cycle arrest by transcriptionally activating the *p21* (*CDKN1A*), *14-3-3*σ** (*SFN*), and *GADD45*α** genes, whereas p53-dependent apoptosis is triggered by turning on the synthesis of proteins that produce ROS [58, 93] and transactivating different mitochondrial proapoptotic genes as well as death receptors for the synthesis of the corresponding proteins [58, 94]. When p53 (and its target genes) expression levels are below a threshold, they induce cell cycle arrest but not apoptosis; above this threshold, p53 trigger apoptosis [95]. Lowering this threshold with inhibitors of the antiapoptotic Bcl-2 family proteins sensitize cells to p53-induced apoptosis [95]. By damaging cells, chemotherapeutical drugs turn on p53's transcriptional function that upregulates the expression of proapoptotic proteins. Defects in the p53 function or in the apoptotic cascades lead to cell phenotypes resistant to chemotherapy. Apoptosis can also be induced by anticancer agents independently of p53 by either directly modulating the apoptotic machinery or directly interacting with mitochondria [96]. ROS levels might be playing a central role in mediating various forms of nonapoptotic, caspase-independent, programmed cell death either through direct irreversible oxidative damage to key proteins, nucleic acid molecules, and cellular structure or by the activation of prodeath signal transduction programs [97]. In a ROS-dependent nonapoptotic cell death pathway, respiration through the mitochondrial electron transport system (ETS) chain is not essential and ROS is generated through NADPH oxidases [97]. In contrast to the mitochondrial ETS which generates ROS as a byproduct of respiration, NADPH oxidases produce ROS as their primary function [98]. Raise of ROS is characteristic after ionizing radiation, chemotherapy, and targeted therapy treatments [58, 99] and may be killing cancer cells through a nonapoptotic ROS-dependent cell death pathway.

Tumor cells maintain high ATP/ADP as well as NADH/NAD^+^ ratios, so as to assure that ATP levels are never limited; ATP depletion would represent then an unsustainable metabolic stress for tumor cells and it is associated with necrotic death in some cell types [100]. Recently, it was shown that a signaling cascade centered on the proteins RIP1 and RIP3 can trigger cell death by necrosis in many situations, and the RIP1-RIP3 complex appears to promote ATP depletion during necroptosis by impinging on components of the MPT pore [101, 102]. Necrosis was a term used to describe a process of accidental cell death, in which cells swell and burst releasing its intracellular content, whereas necroptosis modernly refers rather to an ordered cell death program different from apoptosis; apoptosis is a caspase-dependent cell death process, whereas necrosis is caspase-independent process [102, 103]. Studies concerning necrosis have focused mainly on cell membrane permeability to propidium iodide (PI) in the flow citometry double staining with annexin-V/PI.

Fingrut et al. [17] by studying the effect of MJ on two clones of a highly malignant B-cell lymphoma (one harboring a normal wild type (wt) *p53* gene, while the other expressed a mutated, inactive p53 protein) (Table 1) reported that MJ was equally toxic to both clones [17]. In contrast, the p53-mutant cells were resistant to radiomimetics (drugs having effects similar to those produced in radiotherapy) and chemotherapeutic drugs. In this cancer cell model, MJ induced mostly apoptotic cell death in the wt *p53*-expressing lymphoma cells, while no signs of early apoptosis were detected in the mutant *p53*-expressing lymphoma cells that were killed by MJ through a p53-independent, nonapoptotic cell death mechanism. However, other cancer cell types can undergo apoptosis with or without functional p53, and as we will see, MJ can induce apoptotic and/or nonapoptotic cell death in wt p53 as well as in TP53 mutated cells (Table 1). Nonapoptotic cell death could be the consequence of the severe bioenergetic damages induced by the direct interaction of MJ with mitochondria, leading to increased ROS levels, inhibition of ATP synthesis, and ATP depletion [20, 30]. Jasmonates, including MJ and some of its analogs, have been found to be strongly proapoptotic *in vitro* in different types of cancer cells, by both the extrinsic and the intrinsic pathway (Table 1), with no or little cytotoxicity to normal cells [15, 25, 36, 37, 44, 45]. Thus, MJ induced the expression of TNFR1 in human breast MCF-7 and MDA-MB-435 cells [25, 74] and in hormone-independent human prostate PC-3 and DU 145 cancer cells promoting apoptosis by the extrinsic pathway in these cells [48]. TRAIL (TNF-related apoptosis inducing ligand) is able to induce apoptosis in prostate cancer cell, yet overexpression of antiapoptotic proteins and inhibition of proapoptotic proteins results in diminishing the TRAIL-mediated apoptosis pathway. TRAIL has emerged as a potent anticancer agent in laboratory-based studies and preclinical trials, and JAs have been found to sensitize cancer cells to TRAIL-mediated apoptosis [32] (Table 3). J7, a synthetic analogue of MJ, enhanced TRAIL-mediated apoptosis through upregulation of ROS levels in human hepatoma HepG2 cells; apoptosis was induced via Bid cleavage, downregulation of X-linked inhibitor of apoptosis protein (XIAP), cellular inhibitor of apoptosis-1 (cIAP-1), B-cell lymphoma-extra large (Bcl-xL), and activation of caspases [35]. Other examples of MJ-mediated apoptotic and/or nonapoptotic cell death in different cancer cell types (Table 1) are as follows: (1) MJ induced ROS production in A549 lung adenocarcinoma cells, activating Bcl-2-associated-X-protein (Bax) as well as Bcl-2-Like-1 (Bcl2L1) proapoptotic proteins in these cells [36]; (2) MJ activated caspase-3 in leukemia Molt-4, prostate SK28, and LNCaP cancer cells [15]; (3) MJ inhibited the antiapoptotic Bcl-2 protein and enhanced proapoptotic proteins in radio-resistant human prostate cancer cell line PC-3 [104]; (4) MJ suppressed the proliferation of the neuroblastoma cell lines SH-SY5Y, SK-N-SH and BE(2)-C through a strong apoptosis-inducing effect, as measured by annexin VFITC/PI; MJ treatment also downregulated the expression of the antiapoptotic proteins XIAP and survivin [44, 45]; (5) MJ-mediated cell death was observed on cervical cancer and myeloma cells and it was reported to be due to mixed characteristics of apoptosis and necrosis [26, 105]. So far, studies showing MJ inducing the RIP1 signaling cascade have not been reported; however, it will be important to deep into these analyses to clear distinguish between MJ-induced apoptosis and/or necrosis.

### 2.3. Methyl Jasmonate Induces the Production of Reactive Oxygen Species (ROS) Responses in Some Cancer Cell Types Leading Also to Cell Death

Intracellular ROS levels regulate cell cycle activity, cell metabolism and survival, cell proliferation, and apoptosis [53–58]. Stressful conditions produce a transient excess of intracellular ROS in normal tissues; however, under chronic pathological and oxidative stress conditions, ROS is continuously produced in excess and persistently, causing damage to DNA, cell membranes, lipids, proteins, and inducing apoptosis; to control and avoid oxidative damages, cells counteract excess ROS by enzymatic (superoxide dismutase (SOD), glutathione peroxidase (GPx), and catalase) and/or nonenzymatic (glutathione, thoredoxin) antioxidant elements [99, 106]. Controlling ROS is central to intracellular redox signaling and homeostasis; on the other hand, uncontrolled/unbalanced ROS production modifies intracellular redox leading to apoptosis [58, 82, 99, 106]. ROS can be generated by different biological systems: (1) direct or indirect extracellular stimulation of plasma membrane-bound NAD(P)H oxidases (NOX) can generate intracellular H_2_O_2_ [57, 107–110]; (2) biochemical pathways associated to fatty acid oxidation, and lipoxygenases activities generate ROS as normal byproducts of these activities [111]; (3) ATP generation by oxidative phosphorylation (OxPhos) in mitochondria is accompanied by the production of ROS as a consequence of electron leakage from the electron transport chain; mitochondria are considered to be major sources of ROS in mammalian cells particularly under a variety of stressful conditions [82, 99]. Superoxide (O_2_^−^) is mainly produced from complexes I and III and it is rapidly dismutated to H_2_O_2_ and oxygen by SOD [112]. H_2_O_2_ serves both as a toxic oxidant and as an essential signaling molecule regulating cellular biological processes [113]. A strong positive correlation exists between mitochondrial membrane potential (Δ*ψ*_*m*_) and ROS production [106, 114, 115]. Mitochondria produce more ROS at high membrane potential, and an increase in the Δ*ψ*_*m*_ produced either by a closure of MPT pore or by inhibition of the ATP synthase is associated with increased ROS production. However, in cases of mitochondrial dysfunctions, low Δ*ψ*_*m*_ and decreased activity of the respiratory chain are accompanied by a simultaneous increase in ROS [116, 117]. It has been reported that cancer cells have higher ROS content compared to normal cells [118, 119] probably due to abnormal respiration because of dysfunctional mutations in mitochondria [120], but they also have increased contents of antioxidants (SOD, catalase, glutathione, thioredoxin, etc.) to counteract ROS [58, 121]. Tumor cells cannot tolerate excessive high ROS levels [118, 122] and as a matter of fact, radiation therapy, chemotherapies, and targeted therapies induce apoptosis and kill some cancer cells by generating excess ROS [99, 106, 123, 124]. Thus, inducing higher levels of ROS in cancers cells can be exploited as an effective selective strategy to kill tumor cells over normal cells [119]. However, prolonged treatment with the same drug reduces ROS levels in cancer cells [124], indicating that when they become drug-resistant, tumor cells have lower ROS content than drug-sensitive cancer cells. Evidence suggests that drug-resistant cells have a higher expression of catalase at the plasma membrane that keeps reduced ROS levels [121].

In plants, MJ-mediated damage to mitochondria is followed by a rapid production of H_2_O_2_ [125]. H_2_O_2_ is used as a second messenger for the induction of genes and products related to defense against both herbivores and pathogens [126]; in addition, H_2_O_2_ induces apoptosis and senescence [127, 128]. On the other hand, it has been signaled that the MJ-induced production of H_2_O_2_ in plants was prevented by inhibitors of NAD(P)H oxidase [128, 129] indicating another mechanism of MJ-mediated production of ROS and a major role for this enzyme in MJ-induced ROS responses. MJ induces also an endogenous H_2_O_2_ response in some cancer cell lines (Table 1) [19, 36, 38, 130–132]. In A549 cancer lung cells, the H_2_O_2_ response induced by MJ increased the expression of the proapoptotic Bax and Bcl-Xs (although not being that of the antiapoptotic Bcl-2 and Bcl-xL proteins) and this led to apoptosis; this event was inhibited by catalase (a specific inhibitor of H_2_O_2_) [36]. MJ increased the intracellular levels of H_2_O_2_ and superoxide (O_2_) ions in human C6 glioma cells *in vitro* and induced the expression of heat shock protein 72 (HSP72), a negative regulator of H_2_O_2_-induced oxidative stress, in these cells *in vitro*; MJ-induced HSP72 expression was blocked by ROS inhibitors [130]. Elevated ROS generation was observed in response to both MJ and JA levels in the acute myeloid leukemia HL-60 and KG1a cell lines; but other factors, like the MJ-mediated generation of mitochondrial SOD rather than ROS were determinant for apoptosis or differentiation in these cells [132]. Suppression of MJ-induced apoptosis by antioxidants like N-acetylcysteine (NAC) and catalase, but not by inhibitors of hydroxyl radicals and superoxide ions, indicates that H_2_O_2_ is nonetheless one of the important factors involved in MJ anticancer effects [19].

### 2.4. By Dissociating Hexokinase (HK) from the Voltage Dependent Anion Channel (VDAC) on the Outer Mitochondrial Membrane, MJ Dissociates Glycolysis from Oxidative Phosphorylation Causing Severe Bioenergetic Deregulations in Cancer Cells

Metabolism and cell growth are two cellular processes that are tighly linked and regulated [133]. The classic bioenergetics phenotype of cancer cells of enhanced glycolysis under aerobic conditions was described by Warburg [134]. However, the “Warburg effect” does not necessarily imply mitochondrial dysfunction [135]. To increase glycolysis, cancer cells upregulate the transcription of genes involved in the glycolytic pathway (i.e., glucose transporters, glycolytic enzymes, etc.). This metabolic reprogramming allows increased rate of ATP production, synthesis of lipids, and a new redox balance. Cancer cells actually use a combination of both glycolysis and mitochondrial respiration to produce energy, and they may vary in regards to the preferential use of these pathways, being in some cases either more glycolytic/less oxidative or less glycolytic/more oxidative, depending on the prevalent normoxic or hypoxic environmental conditions and their capacities to express adequate levels of oncogenes and tumor suppressor gene products for cell growth [136–139], or when either glycolysis or OxPhos is inhibited, in whose case there can be partial compensation by the other metabolic pathway [17]. Shifting the balance between those two processes with glycolytic and/or mitochondrial enzyme inhibitors might represent an interesting way to look for new anticancer agents.

The first step in the metabolism of glucose is catalyzed by the enzyme hexokinase (HK) which by phosphorylating glucose makes it negative, sequestering it into the cytoplasm in the form of glucose-6-phosphate (G6P). In normal tissues, this crucial step is catalyzed by four different HK isoforms (HK1, HK2, HK3, and glucokinase) indicating that regulation of glucose phosphorylation can vary in different tissues under different condition [140]. HK1 and HK2 have overlapping tissue expression, but different subcellular distributions, with HK1 associated mainly with mitochondria and HK2 associated with both mitochondrial and cytoplasmic compartments [141]. HK1 is widely and constitutively expressed, whereas HK2 predominates during embryogenesis and is not widely expressed in adult tissues but only in a limited number of normal adult tissues [141]; their different subcellular distributions and kinetic properties reflect their different metabolic roles [141, 142]. Under physiological conditions, HK1 is predominantly bound to the outer mitochondrial membrane where it primarily channels glucose toward glycolysis [140], whereas HK2 is mainly soluble and controls glycogen formation [140, 141]. In malignant tissues, HK1 and HK2 isoforms are glycolytic and overexpressed (HK1 more than HK2) (http://www.proteinatlas.com/) [143] and are tightly bound to mitochondria. HK1 and HK2 bind through their N-terminal hydrophobic regions to the voltage-dependent calcium channel isoform1 (VDAC1), the prevalent pore protein in the outer mitochondrial membrane [24, 82, 143–146]. VDAC1 is also overexpressed in cancer cells [147], and the binding of HK to VDAC1 on the outer mitochondrial membrane is a fundamental aspect of the aerobic glycolytic metabolism of cancer cells [82, 135, 147, 148]. HK/VDAC association appears to protect tumor cells from mitochondrial outer membrane permeabilization (MOMP), which marks a point of no return leading to cell death [149]. VDAC transports ADP and inorganic phosphate (P_i_), the substrates needed for the production of ATP, into the mitochondria; it also transports ATP out of the mitochondria into the cytoplasm and facilitates the availability of ATP to HK2 [143, 150, 151]. A continuous supply of glucose to HK maintains this activity at a constant level and controls the flux through the mitochondrial electron transport chain, influencing mitochondrial ROS production [152]. HK binds through hydrophobic interaction to VDAC1, the prevalent pore protein in the outer mitochondrial membrane [24, 82, 143–146], thereby assuring and gaining a preferential access to ATP [82, 150, 153]. Translocation of HK2 from the cytosol to the mitochondria is regulated through phosphorylation of HK by Akt [141]; HK2 only binds VDAC after phosphorylation by Akt [151, 152]. Glycogen synthase kinase 3 beta (GSK3*β*), a major target of Akt kinases, phosphorylates VDAC [154–157] and dissociates HK [156]. HK2 bound to mitochondria enhance its affinity to ATP and is less sensitive to enzymatic inhibition by the G6P product [140, 141, 158]. HK2 can produce G6P from cytoplasmic glucose and mitochondrial ATP without regulatory restraints, even under hypoxia, supporting rapid cell growth [142, 143, 159–161]. HK1 and HK2 are more tightly bound to VDAC in cancer cells than in nonmalignant cells [156, 162, 163]. HK/VDA1 interaction is relatively stable for HK1, while it is dependent upon G6P levels for HK2 [140, 141]. The higher affinity of mitochondrial HK2 for glucose, ATP, and VDAC1 represents a high advantage for cancer cells survival and growth, conferring to them an aerobic glycolytic phenotype underlying the Warburg effect [82, 135, 138, 142, 147, 148, 150, 160]. Differential interactions of HK1 and HK2 with mitochondria may underlie different glycolytic profiles in cancer cells [164, 165]. As rate-controlling enzymes in the glycolyic pathway, HKs represent important targets for anticancer therapies and drug development; HK inhibition induces cancer cells death and it is reportedly successful in the treatment of cancer [166–169]. In addition, the association between HKs and VDAC provides another therapeutic target. Peptides as well as small molecules that disrupt the interaction between HK and mitochondria can selectively kill tumor cells both *in vitro* and *in vivo* [149, 169].

**Figure 2:**
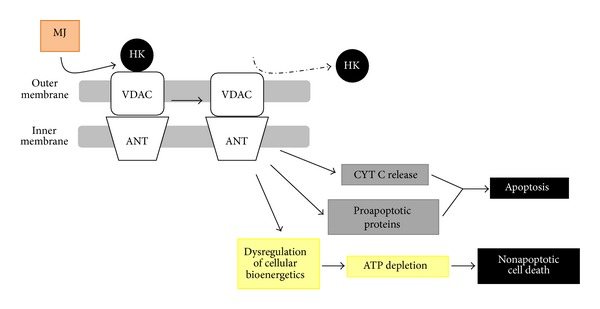
Effect of methyl jasmonate (MJ) on the mitochondrial bioenergetic metabolism of cancer cells. MJ dissociates HK2 from VDAC1 on the outer surface of mitochondria. As an immediate consequence, glycolysis dissociates from oxidative phosphorylation (OXPHOS) and a series of rapid events that occur, such the PTPC opening, mitochondrial membrane permeability deregulation and swelling, decrease of Δ*ψ*_*m*_ and OXPHOS, ATP depletion, cytochrome *c* release, induction of proapoptotic proteins, and cell death through apoptotic and nonapoptotic pathways.

One of the major findings concerning MJ is that it was able to detach HK1 and HK2 from VDAC in a time- and dose-dependent manner in the mitochondrial fraction of murine colon carcinoma CT-36 cells, human leukemic Molt-4 and murine BCL1 cells, and murine B16 melanoma tumor cells by specifically binding to HK2, as judged by HK immunochemical, surface plasmon resonance and planar lipid bilayer VDAC-activity analyses, without inhibiting the kinase activity [30]. These authors emphasized that the susceptibility of cancer cells and mitochondria to jasmonates was dependent on HK overexpression and its association to mitochondria [30]. Dissociation of HK2 from VDAC changed mitochondrial membrane permeability, induced cytochrome *c* release, inhibited ATP synthesis, blocked OxPhos, caused a drastic reduction of intracellular ATP levels, and stopped ATP-driven membrane pumps, causing an overall irreversible bioenergetic damage leading mitochondria to swell and burst and to cell death (Figure 2) [17, 20, 30]. The HK2/VDAC disruption event dissociated glycolysis from OxPhos and in glycolytic cells such as, for instance, CaSki cervical [26], and multiple myeloma [105] cells, and MJ treatment induced an increase in lactate production, whereas at the mitochondrial level, cell death by the intrinsic apoptotic and also by nonapoptotic pathways (including necrosis) was promoted [149], probably related to increased ROS levels [163] (Figure 2). On the other hand, MJ treatment induced an increase in phospho(p)-Akt levels in sarcoma cell lines and activation of the PI3K/Akt pathway attenuated the cytotoxic effect of MJ [49]. This effect was blocked by PI3K/Akt pathway inhibitors and by 2-deoxy-D-glucose (2DG) (Table 3) inducing sensitization toward MJ cytotoxicity in a synergistic manner [49].

MJ-induced ATP depletion was independent of pyruvate (an OxPhos substrate) and oligomycin (an OxPhos inhibitor) [17], but it was inhibited by glucose (a glycolysis substrate); nonetheless, 2-deoxy-D-glucose (a glycolysis inhibitor) synergistically enhanced the anticancer effect of MJ [170] (Table 3). The above described MJ-induced bioenergetic effects were observed only in mitochondria isolated from cancer cells, but not in those isolated from normal cells [19, 38, 170], supporting the general idea that MJ interacts with molecular structures on the outer mitochondrial surface of cancer cells that may be exposed in a substantially different manner than in non-cancerous cells (for instance, the HK2/VDAC complex) [24, 30, 149, 163].

### 2.5. Jasmonates Stimulate the MAPK Stress Pathways and This Effect Leads to Differentiation in Some Cancer Cell Types and to Apoptosis in Others

MAPK pathways are critical for converting diverse extracellular stress signals (ROS, mitogens, mediators of inflammation, and other stressors) to biological responses, such as cell growth, survival, proliferation, inflammatory responses, apoptosis, and differentiation [171]. The main MAPK subfamilies of signaling pathways include the stress-regulated c-Jun NH_2_-terminal kinase (JNK), the p38-MAPK, and the extracellular signal-regulated kinase-1/2 (ERK1/2) pathways. ERK1/2 behaves mainly as mitogen-activated proliferation/differentiation factors [172], whereas JNK and p38-MAPK pathways stimulates the AP-1 transcription factor and regulate responses for cell survival and inflammation and mediate cell death resulting from exposure to various stressors stimuli [173–175].

MJ was able to activate MAPK pathways in different cancer cells types (Table 1). (1) In acute human T-lymphoblastic Molt-4 leukemic cells, MJ activated JNK and p38-MAPK stress responses resulting in AP-1 activity; however, this signaling did not mediate the observed apoptotic leukemic cell death [18]. (2) In A549 lung carcinoma cells a similar effect was seen [19, 36]. The effect of MJ in these two cases was independent of JNK and p38 activities, and AP-1 did not mediate the apoptosis effect on these cells and is independent of RNA and protein synthesis [18]. Thus, MJ induced two independent processes in Molt-4 leukemic cells: apoptotic death and a typical MAPK/JNK and p38 stress response [18]. (3) MJ also activated the p38-MAPK and ERK1.2 pathways in melanocytic MDA-MB-435 cells, but not in the human adenocarcinoma breast cell line MCF-7 [25, 74]. (4) MJ activated the MAPK pathway in the human myelocytic leukemia cell line HL-60, but the outcome of this event was cell differentiation rather than apoptosis [41]; the differentiation effect was inhibited by PD98059, a MEK1 inhibitor, confirming the involvement of MAPK/ERK pathway in this MJ-mediate activity [41]. Acute myeloid leukemia (AML) is one of the worst forms of leukemia (AML blasts are immature myeloid cells), because it is largely resistant to chemotherapeutic drugs and other forms of therapy [176]. These cells can be induced to differentiate into normal, functional myeloid cells by different chemicals; unfortunately, many of these chemicals are too toxic to be used clinically (http://www.cancer.org/acs/groups/cid/documents/webcontent/003110-pdf.pdf). MJ (0.4 mM) stopped the growth of these blast cells and promoted their differentiation to normal cells, as deduced from the expression of markers of differentiation such as NBT reduction (for myelomonocytic differentiation), morphological differentiation into granulocytes, and expression of CD14 (monocyte-specific) and CD15 (granulocyte-specific) surface antigens [41]. MJ induced also the expression of the Ca^2+^-binding protein S100P in these cells, reported to be linked to induction of differentiation (Table 1) [42].

### 2.6. Methyl Jasmonate and Some of Its Synthetic Derivatives Showed Anti-Inflammatory, Antiangiogenic, and Antimetastatic Activities

**Figure 3:**
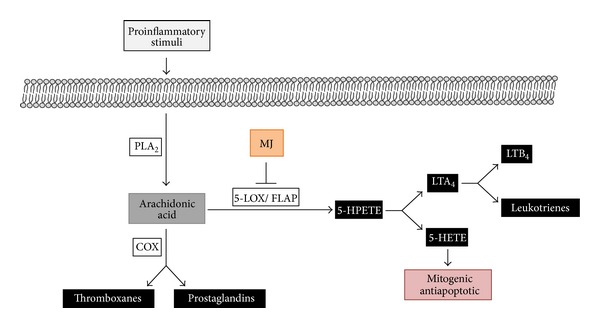
5-Lipoxygenase (5-LOX) pathway in mammalian cells. Methyl jasmonate (MJ) can inhibit this pathway at the 5-LOX level in cancer cells [47]. The multiple consequences of this effect are (1) blocking 5-lipoxygenase-mediated ROS production (lipid hydroperoxides—LOOH); (2) antiproliferative effect linked to inhibition of 5-HETE production; (3) inhibition of leukotrienes production.

The relationship between inflammation and cancer has been widely accepted; particularly, chronic inflammation is a key component where ROS is generated, creating a tumor microenvironment that promotes tumor progression [177]. Relevant inflammation targets in cancer include COX-2, 5-lipoxygenase (5-LOX), and inflammation factors (e.g., inflammatory cytokines: TNF, IL-1, IL-6, and chemokines). The synthetic MJ-derivatives methyl 4,5-didehydrojasmonate (MDDHJ, or J2) and methyl 5-chloro-4,5-didehydro jasmonate (J7), in addition to their antiproliferative and prodifferentiation activity (Table 1), showed a significant anti-inflammatory activities by decreasing nitric oxide (NO), interleukin-6 (IL-6), and TNF-*α* in LPS-activated murine macrophage (RAW264.7) cells [178, 179]. These activities were mediated through the inhibition of the NF-*κ*B pathway and downregulation of miR-155 [178, 179]. On the other hand, MJ may also fights inflammation by blocking the proinflammatory 5-LOX-pathway [47] (Figure 3); 5-LOX is the first enzyme in the 5-lipoxygenase metabolic pathway leading to the synthesis of 5-HETE and leukotrienes, which are harmful proinflammatory substances having direct influence on a number of inflammatory chronic disease processes, including allergic reactions [180], and cancer progression (5-LOX is overexpressed in many aggressive types of cancer, as discussed in the 2.7 section).

The establishment of a tumor generates new blood vessel formation, mainly through hypoxia; angiogenesis plays then an important role in the evolution of both cancer and inflammatory diseases, and it is considered as a potential target for cancer therapy [181]. JAs inhibited angiogenesis at high doses, but the reverse was seen at lower doses in the chorioallantoic membrane (CAM) of chicken embryo [40]. At present, we have not found satisfactory explanations for these results.

Metastasis, rather than the primary tumor, is primarily responsible for anticancer treatment failure, poor quality of life, and death in cancer patients. Cancer metastasis is highly complex and multistep process in nature, involving extracellular matrix degradation, modified tumor cell adhesion, active tumor cell migration, altered tumor cell proliferation, and altered cancer cell survival and angiogenesis; modulation of these processes, particularly the epithelial-to-mesenchymal transition (EMT), in which there is a profound change in transcriptional gene expression [57], enables the cells to escape the primary tumor microenvironment and spread locally/distally establishing a proliferative focus at a secondary site [182]. MJ showed antimetastatic activity on the murine model of highly metastasic and drug-resistant B16-F10 melanoma cells [39]. At low concentrations, MJ was able to reduced cell motility of melanoma cells and suppressed the *in vivo* melanoma growth in the lung; also, the synthetic MJ derivative 5,7,9,10-tetrabromo jasmonate prevented cell adhesion and inhibited lung metastasis at a lower dose than MJ (IC_50_ 0.04 mM *vs.* IC_50_ 2.6 mM) (Tables 1 and 2) [24, 39]. In addition, subcytotoxic concentrations of MJ (0.05–0.2 mM) abolished migration, invasion, and angiogenesis of gastric cancer cells through downregulation of matrix metalloproteinase 14 (MMP-14) [33] (Table 1).

### 2.7. Methyl Jasmonate Can Inhibit the Phase I Drug-Metabolizing Enzyme Aldo-Keto Reductase 1 (AKFR1) that Provides Drug-Resistance in some Tumor Cells

Aldo-keto reductases (AKRs) are soluble cytoplasmic NAD(P)(H) oxidoreductases that reduce aldehydes and ketones to yield primary and secondary alcohols, respectively [183]. Because this reaction permits subsequent conjugation reactions to occur (e.g., sulfation and glucuronidation), AKRs can be referred to as Phase I drug-metabolizing enzymes. The human AKRs can metabolize a vast range of substrates, including drugs, carcinogens, and reactive aldehydes leading to either their bioactivation or detoxification [183]. AKRs are generally monomeric 34–37 kDa proteins present in all phyla; the superfamily consists of 15 families, which contains 151 members (http://www.med.upenn.edu/akr/). Thirteen human AKRs exist that use endogenous substrates (sugar and lipid aldehydes, prostaglandins [184], retinals, and steroid hormones), and in many instances they regulate nuclear receptor signaling [185]. Exogenous substrates include metabolites implicated in chemical carcinogenesis [183]. Human AKRs are highly polymorphic and there is interindividual variation affecting susceptibility to nuclear receptor signaling and chemical carcinogenesis [185]. AKR 1 member B1 (AKR1B1) and AKR 1 member B10 (AKR1B10) are overexpressed in human tumors, such as liver, breast, and lung cancer, and may play critical roles in the development and progression of cancer through carbonyl detoxification, retinoic acid homeostatic regulation, and lipid metabolic control, as well as the activation of tobacco smoke carcinogens [186]. Members of the AKRF1 family are being consistently identified as potential biomarkers for various types of cancer cells [187–189]. Increased mRNA expression of AKR1B1, AKR1C2, and carbonyl reductase (CR) and induction of carbonyl-reducing enzymes AKR1B1 and AKR1C2 can account for drug resistance to some anticancer agents [190–192], for instance, the resistance of human stomach carcinoma cells to daunorubicin [193]. AKR1B1 was also linked to doxorubicin and *cisplatin* resistance in HeLa cervical carcinoma cells because an AKR1B1 inhibitor enhanced the cytotoxic effects of these anticancer agents [194]. All this information clearly indicates that members of the AKR superfamily are emerging as important mediators of cancer pathology [132, 192] and as new targets in cancer drug resistance; therefore AKR1 inhibitors may represent a novel class of antitumor agents.

MJ can bind to members of the AKRF1 family [132] and a correlation has been identified between cell sensitivity to MJ and lower intracellular protein levels of HK2, pVDAC2/3, and AKR1C1 [105].

### 2.8. Many Aggressive Cancer Cell Types Overexpress 5-Lipoxygenase (5-LOX) to Produce the Highly Proliferation-Stimulating Metabolite 5-HETE; MJ Can Inhibit 5-LOX

Lipoxygenases (LOX; linoleate: oxygen oxidoreductase) are a family of monomeric nonheme, nonsulfur iron dioxygenases that catalyze the oxidation of polyunsaturated fatty acids (PUFAs) having a *cis*, *cis*-1,4-pentadiene moiety into lipid hydroperoxides [195]. LOX isozymes add a hydroperoxide group [^−^OOH] at carbons 5, 12, or 15 of AA and are designated 5-, 12-, or 15-lipoxygenases [196]. The major substrates for lipoxygenases in higher plants are linoleic acid (18 : 2) and linolenic acid (18 : 3). In animals, LOXs catalyse the oxygenation of arachidonic (AA) (eicosatetraenoic, C20:4; ETE) acid released from membrane phospholipids by phospholipase A [197] and LOX-derived metabolites participate as signals in several biochemical and physiological processes (http://en.wikipedia.org/wiki/Arachidonic_acid). Arachidonic acid (AA) can be also converted by cyclooxygenase (COX) to prostaglandins (PGs) and other metabolites. LOX converts AA into 5-hydroperoxy-eicosatetranoate (5-HPETE) and then into 5-hydroxy-eicosatetranoic acid (5-HETE) and leukotrienes (B4) (Figure 3). The AA-metabolites derived from COX or LOX reactions modulate many cellular processes and participate not only in various inflammatory reactions, but also in proliferation and apoptosis [195]. Our bodies respond to high levels of dietary-generated AA (triggered by foods rich in *ω*-6 fatty acids and high-glycemic carbohydrates) by increasing the expression of 5-LOX [198]. 5-Lipoxygenase pathway metabolites 5-hydroxy-6,8,11,14-eicosatetraenoic acid (5-HETE) and leukotriene LTB4 stimulates growth and increases survival of cancer cells overexpressing 5-LOX [199–203]. 5-HETE, which enhances cell proliferation and activates also antiapoptotic signaling (Figure 3), has been implicated in human cancer progression. 5-HETE strongly stimulates the growth of hormone-responsive (LNCaP) and hormone-refractory (PC-3) human prostate cancer cells [204, 205]. 5-LOX is rate limiting for the synthesis of 5-HETE and subsequent LTs production; thus, inhibition of 5-LOX would inhibit the generation of these proinflammatory metabolites while promoting cell death [47, 206–211].

Overexpression of 5-LOX has been shown in tissue samples of primary tumor cells [212], in established cancer cell lines and most human aggressive cancers such as breast MDA-MB-231 cells [208], PC-3 prostate cells [47, 204, 205, 207, 213], bladder [15, 214], esophagus [211, 215], pancreas [216], gastric [217], and malignant pleural mesothelial cells [218], as well as lung [219], brain [15, 220], colon [221], hepatocellular carcinoma (HCC) [222], neuroblastoma [223], colorectal [224], glioma cells [225], and Barret's adenocarcinoma [226]; 5-LOX overexpression has been also found in oral carcinoma [227] and canine osteosarcoma [228], all examples strongly suggesting the involvement of 5-LOX in cancer progression. Targeting 5-LOX with specific inhibitors or inhibiting its interaction with the 5-LOX-activating protein (FLAP) has resulted in decreased cell growth and increased apoptosis in lung and breast cancer cell lines [201, 229]. The pharmacological inhibition of 5-LOX by the specific synthetic inhibitor MK886 blocked 5-HETE production potently suppressed tumor cell growth, induced cell cycle arrest, and triggered cell death via the intrinsic apoptotic pathway [204, 230, 231]. Inhibition of 5-LOX activity induced apoptosis in human prostate cancer cells [232] and human pancreatic cancer cells [230] and xenographs [200]. Both the inhibition of 5-LOX activity and the induction of apoptosis in prostate cancer cells were reversed by the addition of 5-HETE [204, 233, 234].

LOX isozymes can be activated and upregulated by UV light exposure, bad fat diets, oxidative stress, ligand-receptor activation (drugs, Fas), ROS-inducing medications, lipid hydroperoxides (LOOH) [198], and their secondary ROS derivatives (free radicals, hydroxides, ketones, aldehydes, etc.) that can exert deleterious effects on membrane lipids (including mitochondrial membrane lipids), thus conforming the first critical step in oxidative stress leading to cell death by the LOX-pathways. Mitochondria that have been damaged by lipid peroxidation decrease their membrane potential (Δ*ψ*_*m*_), increasing cytochrome *c* release, and caspase activation, all these events ultimately leading to apoptosis [195, 235]. LOX-mediated apoptosis implies the following: (1) ROS that (hydroperoxides) induced modifications of membrane properties (exposure of phosphatidylserine, increased levels of cholesterol, and consequently altered Ras expression); (2) modifications of cytoskeleton; and (3) modifications in gene transcription (through nuclear factor NF-*κ*B, poly(ADPribose) polymerase [195]). Apoptosis triggered after an oxidative stress caused by lipoxygenase activation is a common signal transduction pathway shared by animal and plant cells that has been conserved through evolution [235]. The most interesting is the fact that MAPK/ERK, p38MAPK, and MAPK/MEK stress pathways stimulate 5-LOX by phosphorylation [236–238]. 5-LOX phosphorylation does not affect the enzyme catalytic activity but rather regulates its interaction with other cellular components [239, 240]. Thus, for instance, p38 MAPK can phosphorylate 5-LOX at Ser271; phosphorylation at this site is responsible for the stress-induced nuclear export of 5-LOX in CHO-K1 cells (a cell line from Chinese hamster ovary) and HEK293 (human embryonic kidney 293) cells [237], whereas in NIH 3T3 (mouse embryonic fibroblastic) cells, phosphorylation at Ser271 stimulates nuclear localization and subsequently, cellular 5-LOX activity [236].

5-LOX is an important target of JAs since these oxylipids are synthesized in plants via 12-oxo-phytodienoic acid (12-oxo-PDA)—product of an oxidative cyclization of *α*-linolenic acid resulting in cyclopentanones [3, 4]. There are structural and biogenic similarities of 12-oxo-PDA and jasmonates to animal prostanglandins [3, 15, 38]. It is not then a surprise to find that JAs can inhibit the lipoxygenase pathway in animals, MJ being itself a secondary cyclopentanone product derived from of *α*-linolenic acid. MJ significantly inhibited proliferation and induced apoptosis and necrosis in extremely malignant human prostate cancer cells within hours, in dose- and kinetic-dependent manners, showing specific interaction with the 5-LOX pathway [47] (Table 1, Figure 3). The mechanism of inhibition of 5-LOX activity by MJ is still unknown; it is not known if JAs are able to directly interact with the catalytic site of 5-LOX or cause indirect inhibitory effects through interaction with an allosteric site or its regulatory 5-lipoxygenase-activating (FLAP) protein. Relevant to this point is the fact that 5-LOX inhibition may cause cytotoxic and antiproliferative effects in cancer cells independently of suppression of the 5-LOX catalytic activity [239].

## 3. Therapeutic Advantages of Combining Jasmonates with Conventional Anticancer Drug Treatments

Conventional anticancer treatments (surgery, radiation, chemotherapy, and targeted therapies) are all strong inducers of ROS and, as discussed before, ROS excess is a major mechanism for inducing cell death through apoptosis [58, 241]. Excess ROS is highly proinflammatory to cancer neighboring normal cells tissues, being mostly responsible for the painful and aggressive side effects observed in cancer patients [242]. Many phytochemicals have been shown to have anti-inflammatory and also cancer-preventing and anticancer properties (linked to metabolism, proliferation, invasion, angiogenesis, and metastasis activities) showing in addition relatively low toxicity to normal cells [243]. Thus, a rational for a good clinical strategy is combining harsh anticancer treatments with natural anti-inflammatory and cancer-preventing compounds to increase treatment efficacy while lowering drug dose and toxicity [244–246]. However, combination of components having opposite effects, such as for instance antioxidants (anti-ROS), may impair treatment efficacy [57]. Additive and synergic effects may actually be quite advantageous reducing the side effects often seen with single high drug doses treatments in addition to preventing resistant tumor cells to develop [245–247]. Considerable amount of research has been done to identify the relevant molecules that contribute to drug efflux and drug resistance, but the interdependence and cross-talk between metabolic pathways grant cancer cells a high metabolic plasticity, favoring their adaptation to new environmental and stressful conditions [245, 248]. For these reasons, phytochemicals, by being pleiotropic and multitarget, have the great ability to concomitantly modulate multiple metabolic and survival pathways, becoming selectively cytotoxic to cancer cells without inducing toxicity in normal cells [243]; through these properties, phytochemicals can regulate the death receptor pathways and overcome multidrug resistance (MDR) proteins, as documented by multiple examples in laboratory and clinical practice [248].

**Figure 4:**
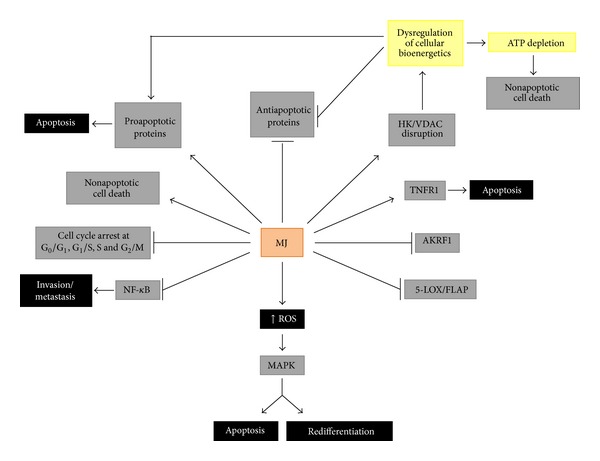
Methyl jasmonate mediated activities in cancer cells. MJ and other jasmonates (JAs) (1) arrest cell cycle, inhibiting cell growth and proliferation; (2) cause cell death by triggering the intrinsic and extrinsic proapoptotic pathways and induce also p53-independent apoptosis and nonapoptotic cell death or necrosis; (3) detach hexokinase (HK) from the voltage-dependent anion channel isoform 1 (VDAC1), dissociating glycolytic and mitochondrial functions, decreasing the mitochondrial membrane potential (Δ*ψ*_*m*_), favoring cytochrome *c* release, causing ATP depletion, and activating proapoptotic and inactivating antiapoptotic proteins; (4) induce reactive oxygen species (ROS), triggering and/or mediating different cellular responses; (5) stimulate MAPK-stress signaling pathways and induce redifferentiation in leukemia cells; (6) inhibit overexpressed cancer cell enzymes such as aldo-keto reductase 1 (AKR1) and 5-lipoxygenase (5-LOX).

By the way of its multiple and different mechanisms of action (Figure 4), MJ may display strong cooperative efficacy with other anticancer agents to induce death in several cancer cells, helping also to overcome multidrug resistance [19, 23, 170]. Combination of MJ with any anticancer drug may have a different rational, synergism being the first and the more important one to take into consideration. If synergism is present, combined MJ-drug formulations may allow for less toxic drug concentrations while keeping an efficient treatment. Chemical structure, physicochemical properties, and molecular mechanisms of action of selected drugs are also important parameters to be considered for combination. MJ is a small molecular weight fatty acid-derived cyclopentanone which is very hydrophobic and is not charged; it would act then better on membranes, and amphipathic vehicles should be considered if combined with hydrophilic drugs. JAs, MJ, and their derivatives have been combined with ionizing radiation, conventional chemotherapy drugs, and also with other phytochemicals, to enhance cancer cell cytotoxicity in anticancer treatments, allowing for lower effective doses while showing enhanced efficacy in inducing death of carcinoma cells (Table 3) [23, 28, 170]. MJ is very selective for cancer cells [17], it is not toxic for normal cells [15, 50], and it induces cell death primarily by triggering mitochondrial perturbation (detachment of HK from the outer mitochondria membrane [30], prooxidant activity, etc.) as deduced from previous discussion. Most aggressive cancers show a glycolytic metabolic profile, and combination of glycolysis inhibitors with OxPhos inhibitors enhances the anticancer properties of this combination. Thus, combination of MJ with 2-deoxy-D-glucose (glycolysis inhibitor) and with four conventional chemotherapeutic drugs resulted in super-additive cytotoxic effects on several types of cancer cells (Table 3). MJ is clearly a mitochondriotoxic anticancer compound, and compounds that directly target mitochondria offer the advantage to induce mitochondrial outer membrane permeabilization independently of upstream signal transduction elements that are frequently impaired in human cancers; in this way, mitochondrion-targeted agents may bypass some forms of drug resistance [96] and cause nonapoptotic cell death.

From Tables 1 and 2 we can infer that MJ acts on a wide gamma of cancer types *in vitro* and *in vivo*, and this property is probably due to its capacity to target multiple metabolic pathways in different cancer cell types, leading to apoptotic and nonapoptotic cell death, as already discussed above. The reported cooperative effects of MJ with other drugs are shown in Table 3: (1) BCNU (carmustine), an alkylating nitrosourea agent with no cytotoxic effect on pancreatic carcinoma cell line MIA PaCa-2 *in vitro* combined with MJ showed enhanced cell death [170]. The underlying molecular mechanisms in this combination are probably related to the DNA damaging action of BCNU on mitochondria [249] rendering the above cells sensitive to MJ which also alter the mitochondrial function [32], thus showing a highly additive cytotoxic effect of two combined mitochondriotoxic agents. This combination acted also synergistically on murine B-cell leukemia (BCL1) cells [170]. (2) MJ (a mitochondriotoxic compound) combined with 2-deoxy-D-glucose (2DG) (an inhibitor of glycolysis) showed synergic/additive effects and cell death on various carcinoma cell types (Table 3) [170]; the synergic effect was probably due to OxPhos/ATP biosynthesis inhibition by MJ and to glycolytic/ATP biosynthesis inhibition by 2DG [17]. MJ enhanced also the toxic effects of taxol [170] much by the same reasons discussed in (1); taxol induces mitochondrial membrane depolarization resulting in translocation of apoptosis-inducing factor (AIF), but not cytochrome *c*, from the mitochondria to the cytosol. (3) MJ enhanced significantly the *in vivo* antileukemic toxic effect of the antibiotic adriamycin (doxorubicin) in a chronic lymphocyte leukemia (CLL) mouse model [170]; adriamycin increases the intracellular levels of ROS, followed by mitochondrial membrane depolarization, cytochrome *c* release, and caspase 3 activation; the IC_50_ value for adriamycin dropped by half when combined with MJ [170, 250]. (4) MJ treatment increased the levels of pAkt in sarcoma cell lines MCA-105 and SaOS-2; however, the treatment of both cell lines with a combination of MJ and small molecular weight PI3K/Akt inhibitors resulted in a synergistic cytotoxic effect [49]. (5) Glucose attenuated the MJ-induced cytotoxicity in these cells; but the treatment with a combination of MJ plus 2DG resulted in a synergistic cytotoxic effect [49]. (6) MJ suppressed the *γ*-radiation-induced expression of Bcl-2 in human prostate cancer cells enhancing the sensitivity of these cells to *γ*-radiation [104]. (7) MJ combined with the monoterpene perillyl alcohol (POH) enhanced the cytotoxicity of *cisplatin* in breast MDA-MB-231 cells and in MDA-MB-435 cancer cells [74, 251]. MJ and POH activate TNFR1 (extrinsic apoptosis pathway), which is further increased by the addition of *cisplatin* [251]. MJ and POH decrease mitochondrial membrane potential (intrinsic apoptosis pathways) inducing cytochrome *c* release, and this effect is further enhanced by *cisplatin* whose cytotoxic activity seems to be largely dependent on the glycolytic metabolism of tumor cells (*cisplatin* would redirect cancer cells to oxidative phosphorylation from the “Warburg effect”) [252]; in this example, the three drugs treatment was more effective than single drug or two drugs together [251]. (8) Survivin (an inhibitor of apoptosis protein (IAP) member) negatively regulates TRAIL-mediated apoptosis in colorectal cancer (CRC) cells but MJ, by downregulating the Wnt/transcription factor pathway, prevented the overexpression of survivin thus sensitizing cancer cells for TRAIL-mediated cytotoxicity [32]. TRAIL is also able to induce apoptosis in prostate cancer cells; however, overexpression of antiapoptotic proteins and inhibition of proapoptotic proteins results in lowering the TRAIL-mediated apoptosis [253]. (9) MJ combined with the Smac peptide (inhibitor of IAPs) showed IAPs-inhibiting synergy in human bladder EJ and T24 cells and human embryonic kidney HEK 293 cells [254]. (10) Hormone-refractory prostate cancer cells are relatively resistant to MJ because the overexpression of IAP proteins decreases the proapoptotic activity of MJ. In these cases, the IAP antagonist SmacN7 (a synthetic peptide comprising the first N-terminal seven residues of Smac) was successfully used to synergically potentiate MJ-mediated apoptosis [255]. The SmacN7/MJ combination induced sensitization to apoptosis through both caspase-9-dependent and caspase-9-independent pathways [255]. (11) MJ worked synergically with *cisplatin* as well as with X-rays irradiation on various cervical cancer cell lines enhancing the cytotoxic effects of these conventional therapies, lowering the effective doses required to inhibit survival of those cells [29]. MJ worked also well with *α*-irradiation enhancing the selective reduction of cell viability and survival of cervical cancer cells effected by *α*-rays [29]. (12) MJ combined with 5-FU in human adenocarcinoma colon HT-39 cells drastically decreased the 5-FU IC_50_ values, which is important to reduce the unwanted side effects usually associated with this drug [31]. (13) MJ synergized also with bortezomib (a proteasome inhibitor) *in vitro* and prolonged survival of immunocompromised mice harboring diffuse lesions of multiple myeloma (MM.1S) cells as compared to vehicle-treated mice [105].

## 4. Concluding Remarks and Hypothesis

As inferred from the many *in vitro* and *in vivo* evidences discussed here, JAs are highly selective towards malignant cells (excepting hormone-independent prostate cancer cells and some sarcoma cell lines which are relatively more resistant to MJ than other cancer cells), not affecting normal cells. Of particular interest is to know that MJ is well tolerated, and no meaningful local or systemic side effects have been detected in animal and humans trials after toxicology and dermatology studies for acute toxicity, skin irritation, mucous membrane (eye) irritation, skin sensitization, phototoxicity, and photoallergy, not being toxic to healthy organisms of different species so far tested and emphasizing the safety of MJ use in humans [50–52]. JAs and particularly MJ have been shown to stimulate and/or to inhibit multiple intracellular pathways and proteins (Figure 4) that may be differently expressed among different cancer cell types. MJ anticancer activities can be summarized as follows: (1) MJ is able to induce reactive oxygen species (ROS), that are subsequent mediators of different intracellular responses; (2) ROS may stimulate the MAPK-stress signaling pathways, inducing ERK-dependent redifferentiation, or apoptosis in leukemia cells; (3) MJ induces cell cycle arrest at different phases in different types of cancer cells, interfering by this way cell growth, proliferation, and migration; (4) MJ promoted the detachment of hexokinase (HK) from to the voltage-dependent-anion-channel isoform 1 (VDAC1) in the outer mitochondrial membrane, dissociating glycolytic and mitochondrial metabolic functions; this event decreases the mitochondrial membrane potential (Δ*ψ*_*m*_), favoring cytochrome *c* release, causing ATP depletion, activating proapoptotic, and inactivating antiapoptotic proteins; (5) MJ induces cell death by triggering the intrinsic/extrinsic proapoptotic, and also nonapoptotic pathways; (6) MJ inhibits metabolic enzymes such as the aldo-keto reductase 1 (AKR1) and 5-lipoxygenase (5-LOX) that favor cancer cells survival and proliferation and that have been found overexpressed in aggressive cancer cells; (7) Jasmonate-mediated anti-inflammatory activities involving downregulation of NF-*κ*B transcription factor activity [178, 179] and inhibition of the 5-LOX pathway; (8) MJ and some of synthetic derivatives inhibit cancer cell invasion and metastasis through downregulation of the NF-*κ*B transcription factor and miR-155 [178, 179]. These pathways may all or partially be expressed in a given cancer cell type at agiven time, and MJ (JAs) can target all of them, causing a catastrophic cancer cellular failure and subsequent cell death (except when inducing redifferentiation), while sparing normal cells. As stated by Elia and Flescher [23], all MJ anticancer mechanisms are not mutually exclusive and may occur concomitantly, so affecting different cancer cells, or can be active in different time frames and concentration ranges [23]. Thus, MJ can probably act simultaneously on all the above targets within the same cell population, and cells may then die by apoptotic and/or nonapoptotic mechanisms, as it was observed by Kniazhanski et al. [26] and Milrot et al. [28] on cervical cancer cells after treatment with MJ (Table 1).

Integrating all the above knowledge we might hypothesize the jasmonates (mainly MJ) mechanisms of action leading to death mammalian cancer cells. By its highly hydrophobic ciclopentanone lipid structure, MJ may easily get across cellular membranes; during this transit, the prooxidant nature of the MJ oxylipin causes an oxidative event that perturbs the lipid bilayer, as reflected by a decrease in membrane fluidity upon contact [25]; this event stimulates NAD(P)H oxidases (NOX) and generates reactive oxygen species (ROS) (mainly H_2_O_2_) [24], both events linked to functions in the MAPK stress pathways [25]; ROS may subsequently act as mediators of other cellular responses as well. The interaction of MJ with the surface plasma membrane might also be responsible for the increased expression of TNFR1 expression and caspase-8 activation observed by Yeruva et al. [25] in some cancer cell types, activating this way the extrinsic pathway of apoptosis. Intracellularly, MJ may bind to lipids and hydrophobic proteins and also interact with subcellular organelles (mitochondria, nucleus, and peroxisomes). A possible mechanism(s) of surface structures discrimination between normal and cancer cells mitochondria by MJ seems to occur, and it is probably related to the overexpression and tight binding of hexokinase(s) (HK) to the VDAC1 pore in the outer mitochondrial membranes (OMM) in cancer cells [24, 30]. By binding to a hydrophobic HK region in the HK/VDAC1 complex on the OMM, MJ induces HK detachment from its VDAC anchor (Figure 2). When this happen, the Bax/Bcl-2 balance is altered, antiapoptotic Bcl-2 proteins are released from the OMM, and proapoptotic proteins occupy their sites inducing the intrinsic way of apoptosis; concomitantly, the mitochondrial membrane potential (Δ*ψ*_*m*_) decreases, cytochrome *c* is released, caspase-3 is activated, mitochondrial ATP depleted, ROS generated, and cell cycle arrested [23, 25, 30], causing a catastrophic energy breakdown and cell death. Nonapoptotic cell death could be the consequence of the severe bioenergetic damages induced by the direct interaction of MJ with mitochondria, leading to increased ROS levels, inhibition of ATP synthesis, and ATP depletion [20, 30]. However, depending on cancer cell type sensitivity to MJ, the extent of mitochondrial damage could be determinant for apoptotic and/or nonapoptotic cell death. In the nucleus, MJ may interact with cell cycle regulatory proteins (cyclins, CDKs, and survivin) inducing cell cycle arrest at different phases in different cancer cell types (Table 1), thus inhibiting cell cancer growth, proliferation, and invasion. MJ-induced downregulation of survivin severely affects cell cycle progression and induces apoptosis [32]. MJ induces TRAIL cytotoxicity, but overexpression of survivin prevents this activity [32]. When targeting several cellular processes, MJ induces ROS production; ROS influences cell cycle progression via phosphorylation and ubiquitination of CDKs and other cell cycle regulatory molecules [57].

The inhibition of multiple cancer cell activities by Jasmonates (MJ) becomes important when we think at cancer as a multifactorial disease that should be better treated through multitarget strategies. Due to their multitarget and pleotropic properties, JAs and particularly MJ are capable of rapidly killing many cancer cell types independently of factors such as cellular mRNA transcription, protein translation [18], and p53 expression [17] leaving untouched normal cells [15]. Due to these properties, MJ may act also as a chemosensitizer to chemotherapics and help to overcome drug-resistance [17, 19]; as seen in Table 3, MJ significantly reduced the IC_50_ values of chemotherapeutic agents with which it was combined. As it has been commented in this and other reviews, JAs can be used alone or in synergic combination with other anticancer drugs and might be seriously considered to be included safely in some of the current anticancer therapies. Finally, the rapidity by which MJ causes damage to cancer cells turns it into a promising anticancer agent of high therapeutic value.

## Figures and Tables

**Table 1:** Effect *in vitro* of natural and synthetic jasmonates on normal and cancer cells.

Jasmonate	(JAs conc range) vehicle	Cell lines	Effects	References
JA, MJ	0.5–3.0 mM/EtOH	Normal mononuclear cells from blood (healthy donors)	No cytotoxicity	[15, 38]
		Acute human T-lymphoblastic leukemia Molt-4 and androgen-responsive human prostate adenocarcinoma LNCap	JA 3 mM: 90% cytotoxicity MJ 0.5 mM: 87.5% cytotoxicity ↑apoptosis (↑caspase-3) ↑necrosis, PTPC opening, arrest at G_0_/G_1_	
		Human melanoma SK-28	JA: ↓proliferation MJ: ↑cytotoxicity ↑cell death	
		Human breast carcinoma MCF-7	JA: ↓proliferation MJ: ↑cytotoxicity ↑cell death	
		Murine lymphoma EL-4	MJ: ↑cytotoxicity	

MJ	3.0 mM no vehicle reported	Acute human T-lymphoblastic leukemia Molt-4	↑p38 ↑JNK ↑AP-1, however, cell death MAPK-independent	[18]

JA, CJ, and MJ	(mM)	Prostate PC-3, HTB-81	↓proliferation	[46]

MJ	0.0–0.4 mM no vehicle reported	Human myeloid leukemia HL-60 cells	↓cell growth ↑MAPK ↑differentiation to mature cells	[41]

MDDHJ (synthetic)	0–250 *μ*M no vehicle reported	Human myeloid leukemia HL-60 cells Monocytoid leukemia U937, THP-1 cells Promyelocytic leukemia NB4 cells Lung adenocarcinoma PC9, PC14 cells	MDDHJ more potent than MJ: ↓cell growth ↑MAPK ↑differentiation to mature cells	[41]

MJ	0.5–5.0 mM	Human lung adenocarcinoma A549	↓proliferation ↑ROS ↑apoptosis (↑Bax/Bcl-Xs ↑caspase-3)	[36]

JA, CJ, and MJ	0.5–3.0 mM/EtOH	Acute human T-lymphoblastic leukemia Molt-4 cells	Molt-4: ↑mitochondrial membrane depolarization ↑cyt *c* release ↑swelling ↑cell death	[38]
		Lymphocytes from CLL patients	CLL cells: ↑mitochondrial membrane depolarization ↑cytotoxicity	
		Liver carcinoma Hep 3B cells	Hep3B: ↑mitochondrial membrane depolarization (PTPC mediated) ↑cyt *c* release ↑swelling ↑cell death	
		Human fibroblast 3T3 cells (nontransformed cell line) Normal blood	Nontransformed 3T3 cells: no cytotoxicity Normal lymphocytes: no cytotoxicity	

JA, MJ	0.25–3.0 mM/EtOH	B-lymphoma clone 29M6.2 (wild type p53) B-lymphoma clone	wt p53 cells: ↑apoptosis mutant p53: ↑nonapoptotic cell death	[17]
		29M6.10 (mutant p53, resistant to treatment)	MJ: ↑~90% ATP depletion in both cell types 2DG, high Glc, but not pyruvate: ↓ATP	

CJ, MJ	0.5–2.5 mM	Nonsmall cell lung cancer lines A549 and H520	↓proliferation, cell cycle arrest at G_2_/M phase ↑p38 and ERK1/2 phosphorylation ↑Bax↑p21 ↑caspase-3	[37]

MJ	0.5–2.0 mM	Hormone-refractory prostate adenocarcinomas PC-3, DU-145	↓proliferation ↑apoptosis ↓5-LOX	[47]

MJ	1–2.6 mM/EtOH—(IC_50_)	Murine melanoma cells B16F10 and B16 COL/R (overexpressing Pgp, MDR)	↓cell motility ↓cell growth ↓MDR	[39]

TBrJA (synthetic)	40 *μ*M/EtOH (MJ doses lower than *in vitro*)	Melanoma B16-F10 Breast MCF-7 Pancreas Mia PCA-2 D122, PBL	↑citoxicity (TBrJA ≫ MJ)	[39]

MJ	0.5–3.0 mM no vehicle reported	CT-26 (murine colon carcinoma) B16 (murine melanoma) BCL1 (murine B-cell leukemia) Molt-4 (human T-lymphoblastic leukemia cell line)	MJ (but not JA) detached HK1 and HK2 from VDAC1 in isolated mitochondria from the four cell lines MJ did not inhibit HKs activity; ↓ATP ↑cyt *c* release ↑mitochondria swelling, ↑cell death	[20, 30]

MJ	1.0-2.0 mM	Human neuroblastoma BE(2)-C	Arrest at S-phase ↓cell growth ↓XIAP mRNA ↓survivin mRNA ↑apoptosis	[43]

MJ	0.5–3.0 mM/DMSO	Human breast cancer MCF-7 Human melanocytic MDA-MB-435 cells [74]	Arrest at G_0_/G_1_and S-phase ↓ membrane fluidity ↑apoptosis: extrinsic (TNFR1, ↑caspase-8); intrinsic [↓Δ*ψ*_*m*_ ↑caspase-3 (only MDA-MB-435)]	[25]

CJ, MJ	2.0 mM/DMSO	Hormone-independent prostate PC-3, DU-145 cells	Cell cycle arrest ↓cell growth ↑apoptosis (↑TNFR1, ↑caspase-3)	[48]

JA, CJ, and MJ	1.0-2.0 mM/DMSO	Human neuroblastoma SH-SY5Y	Arrest at G_2_/M phase ↓cell growth ↑apoptosis (↓XIAP ↓survivin) activities: MJ > JA > CJ	[44]
		Human embryonic kidney HEK 293 cells	Not affected by MJ	

MJ	1.0-2.0 mM/DMSO	Human neuroblastoma SK-N-SH, BE(2)-C	Arrest at G_0_/G_1_ phase ↓cell viability ↓mRNA of PCNA ↑apoptosis (↓XIAP ↓survivin)	[45]

MJ	0.5–3.0 mM/EtOH	Sarcomas: MCA-105,	↑pAkt (correlates with lower sensitivity to cytotoxicity by MJ)	[49]
		SaOS-2 (resistent to MJ)	MJ + 2DG: ↑cytotoxicity	

MJ	1.0–5.0 mM/EtOH	Cervical cancer SiHA, CaSki, and HeLa cells: having wt p53 Cervical cancer C33A cells (with mutated p53)	↓cell cycle ↑apoptosis through different pathways ↓ATP ↑lactate (in more glycolytic CaSki); PARP cleavage, multiple cell death pathways depending on levels of p53, p21, Bcl-2, and Bax	[26]

JA, MJ	0.25–4.0 mM/EtOH	Acute myelogenous leukemia cells HL-60 and KG1	↑ROS ↑MJ-induced mitochondrial membrane depolarization ↑MJ-induced Mit. SOD ↓AKRC1	[132]

MJ, MDDHJ	0.15 mM/DMSO	Leukemia HL-60 cells	↑Ca^2+^-binding protein S100P ↑differentiation ↑regulator of G-protein signaling-16 (RGS16)	[42]

J7 (synthetic)	IC_50_ 15 *μ*M	Human cervical carcinoma HeLa cells	Cell cycle arrest at G_2_/M phase, ↓Bcl-2 (caspase 9, 3) DNA damage	[27]

J7 (synthetic)	50 µM/DMSO	Human hepatoma Hep3B	↑Bax/Bcl-2 ratio ↑DR5 ↑caspase-8 ↓Bid ↑apoptosis correlated with: ↑caspase-9 ↑caspase-3 ↓XIAP ↓cIAP ↓PARP. Extrinsic/intrinsic/MAPK	[34]

MJ	0.5–2.5 mM no vehicle reported	CD138^+^ tumor cells from MM patients	HK2 release from mitochondria, rapid ↓ATP ↑apoptosis	[105]

MJ	0.25–1.0 mM no vehicle reported	Human colorectal cancer cells CRC	↑TRAIL ↑cyt *c* release ↑caspase cleavage ↓survivin ↓TCF transcriptional activity	[32]

MJ	0.0–2.0 mM/EtOH	Cervical cancer cells SiHa, CaSki, HeLa, and C33A	↑mitochondrial O_2_^−^ (HeLa, CaSki) ↓survivin ↓E6, E7 (HPV) ↑different cell death pathways (independently of HPV)	[28]

J7 (synthetic)	0–50 *μ*M/ DMSO	Human hepatoma HepG2	↑ROS ↑TRAIL-mediated apoptosis (↓Bid ↓XIAP ↓cIAP ↓Bcl-xL ↑caspases)	[35]

MJ	3.0 mM (IC_50_) no vehicle reported	Human adenocarcinoma colon HT-39	Arrest at S-G_2_/M ↑cytotoxicity ↑apoptosis	[31]

JA, MJ	1.0–3.0 mM/DMSO	Canine macrophagic malignant DM62 cells	↓cell growth (MJ > JA) ↑cytotoxicity	[256]

MJ	0.5–0.2 mM/DMSO	Human gastric SGC-7901, MKN-45 cell lines	↓migration ↓invasion ↓angiogenesis ↓MMP-14	[33]

Bcl-2: B-cell lymphoma-2; Bcl-xL: B-cell lymphoma-extra large. Bid: BH3 interacting domain death agonist; cIAP: cellular inhibitor of apoptosis; CJ: *cis*jasmonic acid; 2DG: 2-deoxy-D-glucose; Glc: glucose; J7: methyl 5-chloro-4,5-didehydrojasmonate; JA: jasmonic acid; NSCLC: nonsmall-cell lung carcinoma; Pgp: P-glycoprotein; MDDHJ: methyl 4,5-didehydro-jasmonate; MDR: multidrug resistance; MMP-14: matrix metalloprotease 14; PARP: poly (ADP-ribose) polymerase; PCNA: proliferating cell nuclear antigen; PTPC: permeability transition pore complex; ROS: reactive oxygen species; S100P: protein SP100; TBrJA: 5,7,9,10-tetrabromo jasmonate; TNFR1: tumor-necrosis factor receptor-1; TRAIL: tumor necrosis factor- (TNF-) related apoptosis-inducing ligand; XIAP: X-linked inhibitor of apoptosis protein.

**Table 2:** Effect *in vivo* of natural and synthetic jasmonates derivatives.

Jasmonates	Conc. range/vehicle	Organism/tissue/cells	Effects	References
MJ (oral)	236 mg/kg/lipofundin	Normal C57BL/6 mice	Nontoxic	[15]

MJ (oral)	236 mg/kg/lipofundin	C57BL/6 mice injected i.p. with murine T-lymphoma EL-4	↑survival of treated mice as compared to the inoculated control	[15]

TBrJA	40 *μ*M/EtOH (lower doses than MJ *in vitro*)	Murine melanoma B16-F10	↓lung metastasis	[39]

MJ	1–10 *μ*M/EtOH	Chicken CAM	↓angiogenesis	[40]

MJ	1–10 *μ*M/EtOH	Human endothelial cells (HUVEC)	↓COX-2/PGE_2_ pathway	[40]

MJ (i.p.)	1 g/kg in 0.1 mL vegetal oil, i.p.	Multiple myeloma- (MM.1S-) inoculated in nod/scid mice	↑survival of treated mice as compared to the inoculated control	[105]

MJ (topical)	1 g/mL oil	Application on cancerous and precancerous human skin lesions	No local or systemic side effects; 3/8 patients with positive responses	[51]

CAM:chorioallantoic membrane of chicken embryo; HUVEC: human umbilical vein and endothelial cells; TBrJA: synthetic 5, 7, 9, 10-tetrabromo jasmonate.

**Table 3:** Effect *in vitro* and *in vivo* of methyl jasmonate (MJ) combined with other anticancer agents.

Jasmonate	Drug conc. range/vehicle	Model	Effects	References
(1) MJ + BCNU (nitrosourea) *in vitro*	MJ: Fixed conc.	Pancreatic MIA PaCa-1	Mitochondriotoxic synergic cytotoxicity	[170]
(2) MJ + 2DG, adriamycin, taxol, BCNU or *cis*platin *in vitro*	MJ: 0.5–2.0 mM/EtOH Taxol, *cis*platin: 1–10 µg/mL	CT26, DA-3, GTRAMP C1, MCF7, MIA PaCa-2, D122, and BCL1	Strong cooperative effects of MJ + 2DG and MJ + other drugs	
(3) MJ + adriamycin *in vivo*	MJ i.v.: 20–150 mg/kg dissolved in lipofundin. Adr (DOX) i.p.: 4 mg/kg	Balb/c mice injected i.p. with 1 × 10^4^ chronic BCL1 cells lymphocyte leukemia cells	MJ + Adr: significant prolonged survival effect	

(4) MJ + PI3K/Akt inhibitors *in vitro*	MJ: 0.5–3.0 mM EtOH/DMSO	Sarcoma MCA-105, SaOS-2	↓MJ-induced activation of Akt ↑synergic cytotoxicity	[49]
(5) MJ + 2DG *in vitro*	MJ: 0.5–3.0 mM EtOH/DMSO	Sarcoma MCA-105, SaOS-2	2DG: ↓pAkt, ↓MJ-ind. pAkt MJ + 2DG: ↑synergic cytotoxicity	

(6) MJ + irradiation *in vitro*	0.5–2.0 mM DMSO	Irradiated prostate PC-3 (radiation induces Bcl-2 expression)	↓radiation-induced Bcl-2 ↑radiation sensitivity PC-3 ↑caspase-3	[104]

(7) MJ and/or POH and/or *cis*-platin (CP) *in vitro*	Both tested at IC_20_	Human MDA-MB-435 [74]	MJ + POH: ↑TNFR1 ↓Δ*ψ*_*m*_ ↑cytotoxicity; cell cycle arrest at G_0_/G_1_↑apoptosis + CP: all effects enhanced	[251]
		Human breast MDA-MB-231	↑apoptosis	

(8) MJ + TRAIL *in vitro *	MJ 0.5 mM + TRAIL (100–200 ng·mL^−1^)	CRC cancer cells	↓survivin (IAP) ↓Wnt/TCF pathway ↑TRAIL-induced apoptosis ↑caspase activity	[32]

(9) MJ + Smac *in vitro*	MJ: 0.5–2.0 mM DMSO	Human bladder cancer EJ, T24	Synergy: ↑IAPs-bound caspase 3 ↑apoptosis	[254]
		Human embryonic kidney HEK 293	No cytotoxicity	

(10) MJ + Smac7N (IAP antagonist) *in vitro*	MJ: 0.5–2.0 mM DMSO	Hormone-independent prostate DU-145, PC-3 Human proximal tubular epithelial HK-2 cells (overexpressing IAPs)	Smac7N: ↑MJ-induced apoptosis by caspase-9-dependent (intrinsic) and independent (extrinsic) pathways	[255]

(11) MJ + *cis*platin (0.1–0.5 *μ*M) MJ + X-rays (0.25–3 Gy) MJ + *α*-rays	MJ: 0.1–1.0 mM EtOH	Cervical cancer cells SiHa, CaSki, HeLa, and C33A	↓ viability ↓survival ↓IC_50_ radiation dose	[29]

(12) MJ + 5-FU *in vitro *	0.5 mM MJ/1 h, then + 5-FU	Human adenocarcinoma colon HT-39	↓IC_50_ 5-FU (5 → 2.5 mM)	[31]

BCNU: 1,3-bis-(2-chloroethyl)-1-nitrosourea; 2DG: 2-deoxy-D-glucose; 5-FU: 5-fluorouracile; IAP: inhibitors of apoptosis; MM: multiple myeloma; POH: perillyl alcohol; smac: second mitochondria-derived activator of caspases; Smac7N: a peptide that contains the N-terminal seven residues of smac; TNFR1: tumor-necrosis factor receptor-1; TRAIL: tumor necrosis factor- (TNF-) related apoptosis-inducing ligand.
